# Kin selection as a modulator of human handedness: sex-specific, parental and parent-of-origin effects

**DOI:** 10.1017/ehs.2024.24

**Published:** 2024-08-27

**Authors:** Bing Dong, Silvia Paracchini, Andy Gardner

**Affiliations:** 1 School of Biology, University of St Andrews, Dyers Brae, St Andrews KY16 9TH, UK; 2 School of Medicine, University of St Andrews, North Haugh, St Andrews KY16 9TF, UK

**Keywords:** Evolution, game theory, lateralisation, inclusive fitness, genomic imprinting

## Abstract

The frequency of left-handedness in humans is ~10% worldwide and slightly higher in males than females. Twin and family studies estimate the heritability of human handedness at around 25%. The low but substantial frequency of left-handedness has been suggested to imply negative frequency-dependent selection, e.g. owing to a ‘surprise’ advantage of left-handers in combat against opponents more used to fighting right-handers. Because such game-theoretic hypotheses involve social interaction, here we perform an analysis of the evolution of handedness based on kin-selection, which is understood to play a major role in the evolution of social behaviour generally. We show that: (1) relatedness modulates the balance of right-handedness vs. left-handedness, according to whether left-handedness is marginally selfish vs. marginally altruistic; (2) sex differences in relatedness to social partners may drive sex differences in handedness; (3) differential relatedness of parents and offspring may generate parent–offspring conflict and sexual conflict leading to the evolution of maternal and paternal genetic effects in relation to handedness; and (4) differential relatedness of maternal-origin vs. paternal-origin genes may generate intragenomic conflict leading to the evolution of parent-of-origin-specific gene effects – such as ‘genomic imprinting’ – and associated maladaptation.

## Graphical abstract

**Figure:**
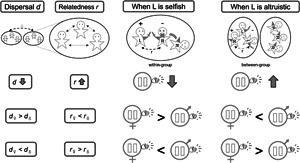

**Social media summary:** We investigate how social interaction between kin affects the balance between right-handers and left-handers in humans.

## 1. Introduction

Most humans show a preference for – or a difference in proficiency of – one hand over the other for a range of tasks (McManus, 2019; Papadatou-Pastou et al., 2020). The frequency of left-handedness in humans is estimated at 10.6%, fairly stably across regions and populations, and is somewhat higher in males (11.6%) than in females (9.5%) (Papadatou-Pastou et al., 2020). Twin studies (Medland et al., 2009) and family studies (Jordan, 1911; Ramaley, 1913; Lien et al., 2015) show that handedness is heritable, with additive genetic effects appearing to explain around 25% of the variance (Medland et al., 2009). Genome-wide association studies (GWAS) have identified 41 loci influencing handedness and explaining around 6% of the heritability (Cuellar-Partida et al., 2021). A recent whole exome sequencing study using the UK Biobank has suggested an association between mutations in the *TUBB4B* gene and left-handedness and estimated that the heritability of left-handedness owing to rare coding variants to be 0.91% (Schijven et al., 2024). Left-handedness has been linked to some psychiatric disorders, such as autism spectrum disorders (ASD) (Markou et al., 2017), schizophrenia (Hirnstein & Hugdahl, 2014) and dyslexia (Abbondanza et al., 2023) and there is an overlap among genes underlying these conditions, brain asymmetries and handedness (Papadatou-Pastou et al., 2020).

Although many taxa exhibit some form of lateralisation (Rogers, 1980; Ocklenburg & Güntürkün, 2012), of which handedness is just one form, these typically involve roughly equal numbers of left-sided and right-sided individuals, and so the strong population bias towards right-handers is peculiarly human (Frayer et al., 2012; Caspar et al., 2022). Language processing is also a typically human skill and is highly lateralised, presenting a left hemisphere dominance in most individuals. However, right hemisphere dominance for language can be observed more frequently in left-handers (Knecht et al., 2000; Mazoyer et al., 2014). Similarly, atypical lateralisation for other functions is more likely to be observed in left-handers than right-handers (McManus, 2022). Understanding how the low but substantial frequency of left-handedness is maintained may therefore serve to illuminate the role of hemispheric specialisation underpinning skills that might have driven human evolution.

The relative stability – with slight variations – of the ~10% incidence of left-handedness in human populations through time (Coren & Porac, 1977; Frayer et al., 2012; but see McManus & Bryden, 1992; McManus, 2009; McManus et al., 2010) and across regions (Papadatou-Pastou et al., 2020) has given rise to the suggestion that left-handedness is maintained by negative frequency-dependent selection, and this has motivated the development of a number of evolutionary game-theoretic hypotheses to explain the phenomenon (Raymond et al., 1996; Ghirlanda et al., 2009; Abrams & Panaggio, 2012; Schaafsma et al., 2012; Faurie & Raymond, 2013). As an illustrative example, the ‘combat hypothesis’ suggests that left-handers suffer a basic disadvantage (Schaafsma et al., 2012; Zickert et al., 2018; Papadatou-Pastou et al., 2020) – e.g. perhaps owing to disruption of typical brain lateralisation – such that natural selection has resulted in them being in the minority, yet also enjoy a compensating advantage when they are sufficiently rare, owing to the element of surprise in combat and similar competitive interactions (Gibbons, 1993; Raymond et al., 1996; Faurie & Raymond, 2013). That is, the rarity advantage explains why left-handedness is present, and the basic disadvantage explains why its frequency is not at 50%. Indirect evidence in support of the combat hypothesis includes higher incidence of left-handers among elite athletes in interactive sports, e.g. tennis, fencing and baseball (Wood & Aggleton, 1989; Goldstein & Young, 1996; Raymond et al., 1996; Loffing, 2017), although the link between left-handedness and reproductive success remains obscure (Groothuis et al., 2013).

These game-theoretic hypotheses centre upon social interaction, whereby an individual's phenotype has an impact upon the fitness of others (the individual's ‘social partners’). Kin selection – the part of natural selection that arises when individuals socially interact with their genetic relatives – plays a major role in the evolution of social interactions across the tree of life (Hamilton, 1964; Frank, 1998; West et al., 2007a). In addition to influencing the overall incidence of traits within and across populations (Sachs et al., 2004; West et al., 2007a), kin selection can explain differences in trait levels between different individuals – such as sex differences (West et al., 2007a; Leedale et al., 2018) – and also modulate evolutionary ‘conflicts of interest’ within families and even within individual genomes – resulting in the evolution of parental genetic effects (Wolf et al., 1998; Richardson et al., 2004; Kuijper & Johnstone, 2016) and parent-of-origin effects, e.g. genomic imprinting (Haig, 2002; Wilkins & Úbeda, 2011). Yet theoretical analyses of the evolution of human handedness have so far failed to consider the possible modulating role of kin selection.

Here we undertake a theoretical investigation of how kin selection may shape the biology of human handedness. First, we show that, at evolutionary equilibrium, left-handedness may be classified either as a ‘selfish’ or an ‘altruistic’ trait, depending on its fitness consequences for the individual and for the individual's social partners, and that the direction of the modulating effect of genetic relatedness depends on which of these two situations applies. Second, we explore how demographic processes such as dispersal modulate the population level of left-handedness at evolutionary equilibrium, via their impact on the degree of genetic relatedness between social partners. Third, we investigate the consequences of sex-biased dispersal, and associated sex differences in an individual's relatedness to social partners, for the evolution of sex differences in left-handedness. Fourth, we determine the consequences of extending genetic control of handedness to the individual's parents, resulting in parent–offspring conflict and sexual conflict and the evolution of parental genetic effects in relation to human handedness. Fifth, we descend to the level of individual genes and investigate the scope for intragenomic conflict between maternal-origin vs. paternal-origin genes and the resulting evolution of parent-of-origin effects – including genomic imprinting – in relation to human handedness.

We conduct our theoretical investigation by means of a qualitative exploration of the logic of kin selection as it applies to left-handedness, and we complement this conceptual approach with an explicitly mathematical illustrative analysis presented in the Supplementary Material. For the purpose of illustration and concreteness, in each case we derive predictions for ‘within-group combat’ and ‘between-group combat’ game-theoretic scenarios, but our analysis applies to any scenario in which an individual's handedness has an impact upon their own reproductive success and that of their genetic relatives. We allow for handedness to be a highly polygenic trait and, although for ease of conceptualisation we will refer to handedness in a binary way, our analysis readily accommodates a spectrum of handedness. Indeed, in contrast to previous studies which have focused on the consequences of these different genetic architectures (McManus & Bryden, 1992; McManus et al., 2013; McManus, 2022), our focus is on how natural selection shapes the handedness phenotype. More generally, rather than giving *post hoc* explanations for existing observations, our major aim is to provide *a priori*, testable, comparative predictions to motivate new empirical research avenues and to develop a theoretical framework within which the results of future analyses can be conceptualised.

## 2. Results

### (a) Kin selection and human handedness

Natural selection adapts individuals as if for the purpose of passing on their alleles to future generations (Hamilton, 1964; Grafen, 2006; West & Gardner, 2013). There are two basic routes through which individuals can accomplish this: first, by promoting their own reproductive success (direct fitness); and second, by promoting the reproductive success of their genetic relatives, who tend to share alleles in common (indirect fitness) (Hamilton, 1964). The part of natural selection that is driven by the impact of individuals on their genetic relatives defines ‘kin selection’ (Frank, 1998). According to Hamilton's (1964) rule, a behaviour that incurs a fitness cost *c* for the actor can nevertheless be ‘favoured’ by natural selection if it provides a sufficiently large fitness benefit *b* to a sufficiently closely related recipient, where the kin-selection coefficient of relatedness *r* describes the statistical association between their genetical traits (i.e. *r* = 1/2 for full siblings, *r* = 1/4 for half-siblings, *r* = 1/8 for first cousins, and so on, in a simple outbred population setting; Frank 1998). Specifically, the behaviour is favoured if −*c* + *rb* > 0. More generally, we can define four types of social behaviour, according to the sign of the fitness effects: traits incurring a cost for the actor and yielding a benefit for the recipient (*c* > 0 and *b* > 0) are ‘altruistic’; traits yielding a benefit for the actor and incurring a cost for the recipient (*c* < 0 and *b* < 0) are ‘selfish’; traits yielding a benefit for both parties (*c* < 0 and *b* > 0) are ‘mutually beneficial’; and traits incurring a cost for both parties (*c* > 0 and *b* < 0) are ‘spiteful’ (Hamilton, 1964; West et al., 2007b).

At evolutionary equilibrium, where natural selection favours neither an increase nor a decrease in the trait (−*c* + *rb*  = 0), then so long as relatedness is positive (*r* > 0) the trait must either be marginally altruistic (*c* > 0 and *b* > 0) or marginally selfish (*c* < 0 and *b* < 0) (Hitchcock et al., 2019). Accordingly, if natural selection acts in a negative frequency-dependent way in relation to human handedness – as suggested by the game-theoretic models (Ghirlanda & Vallortigara, 2004; Ghirlanda et al., 2009; Abrams & Panaggio, 2012) – such that it favours an increase in the incidence of left-handedness when this has dropped below a threshold level and favours a decrease in left-handedness when it has exceeded the threshold, then evolutionary equilibrium is attained when the incidence of left-handedness is at the threshold, and at this point left-handedness is either marginally altruistic or marginally selfish. If left-handedness is marginally altruistic then a higher degree of genetic relatedness between actor and recipient is expected to be associated with a higher incidence of left-handedness at the evolutionary equilibrium, whereas if left-handedness is marginally selfish then a higher degree of genetic relatedness is expected to be associated with a lower incidence of left-handedness.

**Figure 1.**
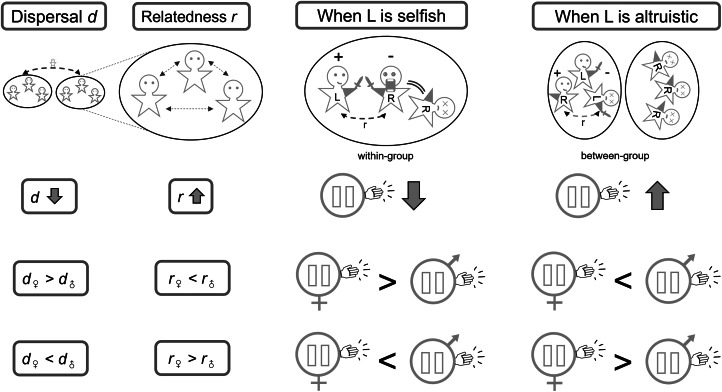
Comparative predictions as to how dispersal affects relatedness between social partners and hence the evolutionarily favoured incidence of left-handedness, depending on the type of social interactions: within-group (selfish) or between-group (altruistic) combat. Lower dispersal leads to higher relatedness, hence low dispersal favours a lower incidence of left-handedness when it is selfish and a higher incidence of left-handedness when it is altruistic. Sex-biased dispersal creates asymmetry in relatedness and hence favours a sex difference in left-handedness.

Taking the combat hypothesis as a purely illustrative example, if we imagine that combat occurs mainly within human groups – i.e. between individuals who are members of the same community and are somewhat genetically related to each other – then the indirect-fitness consequences of enjoying a surprise advantage in combat owing to left-handedness are expected to be negative (because the opponent, who loses out, is a genetic relative). Hence, at equilibrium, this indirect-fitness cost is expected to be exactly balanced by a direct-fitness benefit (owing to improved success in combat outweighing the basic disadvantage of left-handedness). In this scenario, left-handedness is a marginally selfish trait, and kin selection disfavours left-handedness, such that a higher level of relatedness is expected to be associated with a lower incidence of left-handedness (Figure 1 and S3a). Alternatively, if combat mainly occurs between non-relatives in an intergroup-warfare context in which success in combat is associated with a positive indirect-fitness effect owing to the benefits that accrue to the individual's genetically related groupmates, then at equilibrium this is expected to be exactly balanced by a direct-fitness cost (owing to the basic disadvantage of left-handedness failing to outweigh the improved success in combat). In this alternative scenario, left-handedness is a marginally altruistic trait, and kin selection favours left-handedness, such that a higher level of relatedness is expected to be associated with a higher incidence of left-handedness (Figure 1 and S3a).

Genetic relatedness will usually depend on the ecology and demography of the population, and so the above kin-selection logic also yields predictions as to how population processes relate to the evolutionarily favoured incidence of left-handedness. As a concrete example, we consider the rate of dispersal. If individuals have a higher tendency to disperse away from their place of origin and pursue reproductive opportunities within other groups, then this is expected to result in lower relatedness among groupmates. Accordingly, if left-handedness is marginally selfish – as, for example, in the within-group combat scenario – then as the rate of dispersal increases, the degree of relatedness decreases, and hence the evolutionarily favoured level of left-handedness is expected to increase (Figure 1 and S3a). And, in contrast, if left-handedness is marginally altruistic – as, for example, in the between-group combat scenario – then as the rate of dispersal increases, the degree of relatedness decreases, and hence the level of left-handedness is expected to decrease (Figure 1 and S3a). These predictions relate to contemporary and/or historical between-population comparisons and also, potentially, to the dynamics of handedness within a single population across evolutionary timescales in responses to demographic change (see Discussion).

### (b) Sex differences in human handedness

Above, we have shown that the average genetic relatedness between social partners – and the population processes that modulate this – is expected to influence the evolutionarily favoured incidence of left-handedness at a population level. Similarly, inter-individual differences in relatedness to one's social partners – and the population processes responsible for such variation – are expected to drive differences in levels of left-handedness among different subdivisions of the population. In particular, sex-specific demographic processes – such as sex-biased dispersal – may result in a sex difference in the relatedness of social partners, which may therefore favour a sex difference in the incidence of left-handedness. For example, all else being equal, female-biased dispersal is expected to result in relatedness between social partners being lower for woman than for men. Hence, all else being equal, a higher level of left-handedness would be favoured among women than among men if left-handedness is marginally selfish (such as in the within-group combat scenario) and a higher level of left-handedness would be favoured among men than among women if left-handedness is marginally altruistic (such as in the between-group combat scenario). The opposite pattern is expected under male-biased dispersal (Figure 1 and S3b).

In addition to differences in relatedness, the sexes might also differ with respect to the fitness consequences – that is, the benefits and costs – associated with left-handedness. Such fitness differences would also be expected to modulate sex differences in the incidence of left-handedness. For example, if the frequency-dependent advantage of left-handedness when rare applies more strongly to men than to women – as would be expected in the combat scenarios if men engage in combat more frequently than do women and/or if men have more to gain from winning in combat in terms of enhanced reproductive success (Micheletti et al., 2018) – then, all else being equal, the incidence of left-handedness is expected to be higher among men than among women. Note that we need not expect each sex's observed level of left-handedness to correspond exactly to its adaptive optimum; to the extent that the same genes underpin left-handedness in both sexes, sexual antagonism might keep both sexes from attaining their respective optima (Grafen, 2006). More generally, these sex-difference results concern adaptive evolution, and are based upon considerations of female vs. male fitness optima. Accordingly, they neglect non-adaptive sex differences arising, for example, from a greater vulnerability of males to developmental perturbation away from a default phenotype, which has been reported in disorders including ASD (Antaki et al., 2022). Such non-adaptive mechanisms could offer alternative explanations for the higher incidence of left-handedness among males (see Discussion).

### (c) Parental genetic effects in human handedness

Above, we have shown how the evolutionarily favoured level of left-handedness may be modulated by the evolutionary value that individuals place upon the reproductive success of social partners relative to their own reproductive success. This assumes that an individual's own genotype controls the handedness phenotype. If, instead, the handedness phenotype were controlled by the parental genotype – i.e. a ‘parental genetic effect’ (Trivers, 1972, 1974; Wilson, 1980) – then we might expect the evolutionarily favoured incidence of left-handedness to reflect the relatedness valuations made by the individual's parents. More generally, if an individual's predisposition to left-handedness is modulated in part by the individual's own genotype and also in part by the genotypes of the individual's parents then we might expect an evolutionary ‘conflict of interests’ – and associated evolutionary arms race – between parent and offspring (Trivers, 1974), and between the parents themselves (Trivers, 1972), as each party is favoured to move the handedness phenotype closer to their own fitness optimum.

If an individual's handedness phenotype represents a trade-off between the individual's own reproductive success and the reproductive success of the individual's groupmates, then in general terms we expect the individual's parents to favour a balance that is relatively biased towards the groupmates’ reproductive interests and the individual to favour a balance that is relatively biased towards their own reproductive interests, so long as there is relatedness among groupmates (see Supplementary Material §§S1.7 and S2.5 for details). This owes to individuals being genetically identical to themselves and only somewhat genetically related to their offspring. Accordingly, if left-handedness is a marginally selfish trait (as in the illustrative within-group combat scenario) then we expect parents to favour a lower predisposition for left-handedness in their offspring than their offspring would themselves favour. And if left-handedness is a marginally altruistic trait (as in the illustrative between-group combat scenario) then we expect parents to favour a higher predisposition for left-handedness in their offspring than their offspring would themselves favour (Figure 1 and S3c).

Moreover, although both parents are equally related to their offspring they may be differentially related to their offspring's social partners, so that mothers and fathers may favour different dispositions for left-handedness among their offspring. For example, under female-biased dispersal, mothers are expected to be less related to their offspring's social partners than are fathers, and hence more inclined to their offspring having a disposition for left-handedness if this is a marginally selfish trait (as in the illustrative within-group combat scenario) and less inclined to their offspring having a disposition for left-handedness if this is a marginally altruistic trait (as in the illustrative between-group combat scenario). The opposite set of outcomes is expected under male-biased dispersal (Figure S5). Accordingly, considerations of patterns of relatedness and concomitant kin selection yield predictions as to parental genetic effects – including maternal genetic effects and paternal genetic effects – working at cross purposes with the individual's own genome, as well as with each other, in relation to the individual's handedness phenotype.

### (d) Parent-of-origin effects in human handedness

Above, we have shown that sex-specific demography – such as sex-biased dispersal – may generate differences in the relatedness valuations made by mothers and fathers regarding the reproductive success of their offspring vs. their offspring's social partners, resulting in the evolution of parental genetic effects in relation to handedness. Similarly, this relatedness asymmetry can also extend into the offspring's own genome and ignite an evolutionary conflict of interests between the individual's own maternal-origin vs. paternal-origin genes. Such intragenomic conflict in relation to other social traits has been suggested to drive the evolution of parent-of-origin specific genetic effects, including genomic imprinting (Haig, 2002; Gardner & Úbeda, 2017) – and induce vulnerability to a number of associated developmental disorders, e.g. Silver–Russell syndrome and Beckwith–Wiedemann syndrome (Crespi, 2011; Wilkins & Úbeda, 2011).

**Figure 2.**
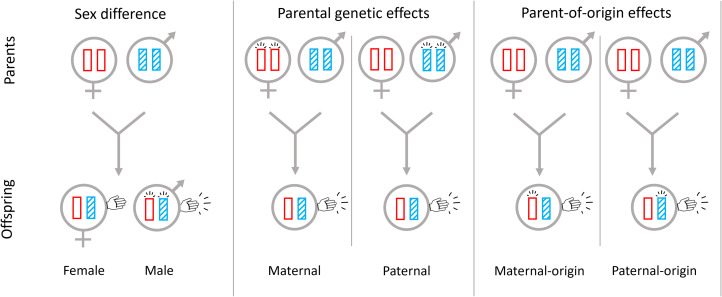
Sex differences, parental genetic effects and parent-of-origin effects at loci underpinning left-handedness. Sex-biased expression of genes can lead to a sex difference in incidence of left-handedness. Expression of parents’ genes can lead to parental genetic effects in relation to left-handedness. Parent-of-origin-specific gene expression can lead to parent-of-origin effects in relation to left-handedness.

For example, if left-handedness is marginally selfish (such as in the within-group combat scenario) then under female-biased dispersal the relatedness between social partners through maternal-origin genes – all else being equal – will be lower than the relatedness through paternal-origin genes, and hence maternal-origin genes are expected to favour a higher level of left-handedness than are paternal-origin genes (Figures 2 and S4). Under male-biased dispersal relatedness will be higher through maternal-origin genes than through paternal-origin genes, and hence maternal-origin genes are expected to favour a lower level of left-handedness than are paternal-origin genes (Figures 2 and S4). Conversely, when left-handedness is marginally altruistic (such as in the between-group combat scenario) then under female-biased dispersal maternal-origin genes are expected to favour a lower level of left-handedness than are paternal-origin genes, whereas under male-biased dispersal maternal-origin genes are expected to favour a higher level of left-handedness than are paternal-origin genes (Figure 2 and S4).

According to the kinship theory of genomic imprinting (Haig, 2002), this form of intragenomic conflict will typically lead to one of the copies of the gene being silenced. Specifically, according to the ‘loudest voice prevails’ principle (Haig, 2002), the two copies of the gene at the affected locus are favoured to adjust their level of expression in opposite directions, such that the one favouring a higher level of left-handedness will act to increase the level of left-handedness while the one favouring a lower level of left-handedness will act to decrease the level of left-handedness, until the gene being favoured to decrease its expression falls silent. At a locus for which an increase in gene expression results in an increase in the level of left-handedness – a ‘left-handedness promoter’ locus – it is the gene that favours a higher level of left-handedness that is expected to remain expressed while the gene that favours a lower level of left-handedness is silenced. And at a locus for which an increase in gene expression results in a decrease in the level of left-handedness – a ‘left-handedness inhibitor’ locus – it is the gene that favours a lower level of left-handedness that is expected to remain expressed while the gene that favours a higher level of left-handedness is silenced. Accordingly, the function of the gene product determines the direction of imprint.

**Figure 3.**
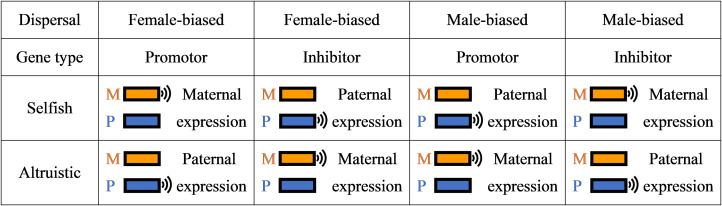
Predictions as to how dispersal pattern and gene function modulate the pattern of genomic imprinting. See Supplementary Material Figure S7 for additional predictions concerning the phenotypic consequences of gene deletions, gene duplications, epimutations and uniparental disomies.

For example, if left-handedness is marginally selfish (e.g. within-group combat), then under female-biased dispersal we expect left-handedness promoters to be maternally expressed and paternally silenced and left-handedness inhibitors to be maternally silenced and paternally expressed, and under male-biased dispersal left-handedness promoters are expected to be maternally silenced and paternally expressed and left-handedness inhibitors to be maternally expressed and paternally silenced. However if left-handedness is marginally altruistic (e.g. between-group combat), then under female-biased dispersal we expect left-handedness promoters to be maternally silenced and paternally expressed and left-handedness inhibitors to be maternally expressed and paternally silenced; and under male-biased dispersal left-handedness promoters are expected to be maternally expressed and paternally silenced and left-handedness inhibitors to be maternally silenced and paternally expressed (Figure 3).

## 3. Discussion

Although game theoretic attempts to explain the evolutionary maintenance of a substantial minority of left-handed individuals in human population fundamentally hinge upon social interaction, and although kin selection is a fundamental driver of social evolution, the possible role for kin selection in modulating the evolution of human handedness has previously been neglected. We have shown how patterns of genetic relatedness – and the demographic processes underpinning these – are expected to shape patterns of human handedness. Specifically, our kin-selection analyses show that: (1) relatedness between social partners – modulated by population processes such as dispersal – is expected to influence the population level of left-handedness in a direction that depends upon whether left-handedness is marginally selfish (as in our illustrative within-group combat scenario) vs. marginally altruistic (as in our illustrative between-group combat scenario); (2) sex-specific demography – such as sex-biased dispersal – can result in sex differences in relatedness to one's social partners, which may go some way to explaining sex differences in incidence of left-handedness; (3) differences in relatedness valuations made by different family members can ignite conflicts of interest between parents and offspring and between an individual's mother and father over their handedness phenotype, driving the evolution of parental genetic effects; and (4) such relatedness differences may even ignite evolutionary conflicts of interest within the individual's own genome, with maternal-origin and paternal-origin genes favouring different handedness phenotypes, which is expected to drive the evolution of parent-of-origin effects – such as ‘genomic imprinting’ – in relation to handedness. By pitching our analysis in a qualitative way we achieve a considerable degree of generality, with our comparative predictions applying across a diversity of genetic architectures. Although factors such as the number of loci underpinning the left-handedness phenotype and the size of human groups will clearly make a quantitative impact on evolutionary outcomes, the basic logic of kin selection applies as generally as the concept of natural selection itself (Gardner et al., 2011).

Our analyses have shown that the degree of genetic relatedness between social partners is expected to modulate the evolutionary equilibrium frequency of left-handedness in the population, with higher relatedness being associated with a lower level of left-handedness when left-handedness tends to benefit the individual at the expense of social partners (selfishness) and a higher level of left-handedness when left-handedness tends to benefit social partners at the expense of the individual (altruism). The degree of relatedness is itself expected to depend on ecological and demographic parameters such as rate of dispersal, with higher dispersal of individuals tending to reduce the extent of genetic relatedness between social partners. At a comparative level, variation in ecological and demographic parameters between different human populations could potentially explain between-population differences in incidence of left-handedness. Variation in ecological and demographic parameters within a single human population over time might also explain temporal differences in the incidence of left-handers, but only insofar as the variation in ecology and demography occurs over a relatively long timescale and the evolutionary fine-tuning of handedness occurs over a relatively short timescale – i.e. so that there is time for adaptation to current conditions to occur before those conditions change. Our analysis offers little quantitative guidance as to the relevant timescales, but the population bias towards right-handedness does appear to have already been in place when hominin lineages diverged from the great apes around 7 million years ago (Uomini & Ruck, 2018; Papadatou-Pastou et al., 2020).

Our analysis also shows that sex-specific selection can give rise to sex differences in handedness. We have shown how sex-specific demographies – such as sex-biased dispersal – may lead to sex differences in relatedness between social partners and hence sex-differences in the level of left-handedness favoured by females vs. males. Whether humans have been characterised by sex-biased dispersal in our evolutionary past, and in which direction, remains a controversial topic: the traditional view is that human dispersal has been female-biased (Ember, 1975), but evidence has also been marshalled in support of dispersal having been unbiased or mixed (Marlowe, 2004). Our use of sex-biased dispersal is merely as an illustration, and the results extend more generally to any ecological and demographic factors that result in sex-differences in relatedness to one's social partners – such as patterns of inbreeding (Wilkins & Haig, 2003). In addition to relatedness, our analysis has emphasised that sex difference in left-handedness might also reflect sex differences in the costs and/or benefits of left-handedness. For example, men are generally understood to engage in – and to benefit from winning – combat more than do women (Micheletti et al., 2018), which could explain a higher incidence in left-handedness on account of a surprise advantage in combat settings. The higher incidence of left-handedness in males could also arise for non-adaptive reasons. One possibility is sexually differential liability thresholds (Khramtsova et al., 2019; Merikangas & Almasy, 2020; Antaki et al., 2022), whereby the number of risk alleles required for an individual to exhibit a minority phenotype is greater for females than males, i.e. the ‘female buffering’ effect. Another is sex-linked inheritance (McManus & Bryden, 1992; Jones & Martin, 2010; but see McManus, 2010), although X-linked left-handedness genes have not been found in the recent sex-stratified GWAS analysis (Cuellar-Partida et al., 2021).

Our analysis shows the potential for parental genetic effects to occur in relation to left-handedness, such that alleles carried by a parent exert an influence on their offspring's handedness phenotype, irrespective of whether the offspring carries the same alleles. These parental genetic effects are expected to arise evolutionarily as a consequence of parents having different interests regarding their offspring's handedness phenotype, and our analysis yields predictions as to patterns of such genetic effects depending on the sex of parent and offspring (see Supplementary Material §§S1.7 and S2.5). Note that we have taken a ‘battleground’ approach (Godfray, 1995) that enables characterisation of the existence and direction of this parent–offspring conflict. A further ‘resolution’ model (Godfray, 1995) would be required to explore more fully the resulting arms race and to determine its evolutionary endpoint – this represents an avenue for future theoretical analysis. Schmitz et al.'s (2022) genomic analyses suggest the existence of parental effects in relation to hand preference, and stronger maternal effects than paternal effects in another multidimensional laterality trait – footedness, when controlling for possible non-paternity cases. However, despite the well-known difference in association between offspring handedness and maternal vs. paternal handedness (Jordan, 1911; Ramaley, 1913), the possibility of parental genetic effects has been neither confirmed nor excluded. Parental genetic effects have been suggested to arise in neurodevelopmental disorders associated with handedness, such as maternal genetic effects in relation to loci associated with ASD – potential loci include *SHANK3* on chromosome 22 and *WBSCR17* on chromosome 7q11 – but these findings have not been replicated (Connolly et al., 2017). The predictions of our analysis therefore offer a new perspective for understanding the role of parental genetic effects in neurodevelopmental disorders.

Finally, our analysis also shows maternal-origin vs. paternal-origin genes within an individual's own genome may come into conflict in relation to their carrier's handedness phenotype, and how this conflict may lead to the evolution of parent-of-origin-specific gene expression. Genomic imprinting is associated with a variety of debilitating disorders, with parent-of-origin-specific clinical effects and non-standard patterns of inheritance that are often predictable in light of the kinship theory (Wilkins & Úbeda, 2011). Our results concerning patterns of imprinting allow us to make predictions as to the effects of a range of different mutational and epimutational perturbations of imprinted loci affecting handedness (Figure S7). For example, a gene deletion at an imprinted locus is expected to have no impact on the phenotype if the gene was to be silenced anyway, but it is expected to have a potentially major impact upon the phenotype if it was to be expressed such that no functional gene product at all will derive from the affected locus (Figure S7). Such effects might often be lethal insofar as they involve disruption to early stages of brain development when left–right asymmetry is usually established. These predictions could potentially enhance our understanding of various neurodevelopmental disorders associated with handedness. A range of neurodevelopmental conditions are associated with an elevated level of left (or non-right) handedness, e.g. dyslexia or developmental language disorders (Abbondanza et al., 2023; Packheiser et al., 2023), schizophrenia (Hirnstein & Hugdahl, 2014) and ASD (Markou et al., 2017). Several loci that are associated with ASD have been suggested to have parent-of-origin effects – with maternally over-expressed components including a region between *LOC391642* and *LOC645641* on chromosome 4 and the *LRRC16A* gene on chromosome 6, and paternally over-transmitted genes including the *STPG2* gene on chromosome 4 and the *TBC1D4* gene on chromosome 13 – but these findings are not replicated (Connolly et al., 2017). Considering novel parent-of-origin effects on complex traits have recently been reported with larger samples and new methods such as the probabilistic approach (Hofmeister et al., 2022), we suggest that parent-of-origin effects might be more widespread than anticipated.

In relation to parent-of-origin effects, we have focused on the ‘loudest voice prevails’ model of the evolution of genomic imprinting (Haig, 2002), which applies here to loci whereby a greater level of gene expression either increases (‘left-handedness promoter’) or decreases (‘left-handedness inhibitor’) the likelihood of the individual exhibiting left-handedness. For loci at which an intermediate level of gene expression yields a right-handed phenotype and deviations in gene expression (in either direction) are liable to yield a left-handed phenotype – in line with the developmental instability hypothesis of handedness (Yeo & Gangestad, 1993) – we might instead expect the gene that favours a greater incidence of left-handedness to exhibit more stochastic expression, i.e. the ‘chaotic voice prevails’ logic of Úbeda et al. (2014). More generally, the kinship theory of genomic imprinting, as it currently stands, predicts genomic imprinting of all loci that experience parent-of-origin conflict, irrespective of the intensity of the conflict. Yet empirical studies suggest that genomic imprinting is quite rare – around 1% of genes in the human genome (Luedi et al., 2007). Clearly, there are additional requirements for a locus to evolve imprinting, and our hope is that through confronting these quite speculative theoretical predictions with empirical data, the theory can be further refined.

The strong population bias in favour of one sidedness type while the other remains a substantial minority appears to be an exclusively human phenomenon. However, lateralisation itself has a taxonomically widespread occurrence. The historical view that lateralisation is unique in humans was disputed in 1970s during a renaissance of lateralisation studies (Güntürkün et al., 2020), and since then lateralisation has been reported across the animal kingdom (Rogers, 1980; Ocklenburg & Güntürkün, 2012). Some species show lateralisation only at individual level, such as paw preference in rodents (Manns et al., 2021), and in cats and dogs (Ocklenburg et al., 2019), turning preferences in insects (Girling et al., 2007) and in fishes (Vallortigara & Rogers, 2005), and eye preference in octopuses (Byrne et al., 2004). While lateralisation at population level seems to be relatively rarer (Vallortigara & Rogers, 2005; Meguerditchian et al., 2013), supporting evidence has steadily accumulated from studies of indoor/captive individuals and from the field (Forrester et al., 2013), including hand preference in non-human primates (Caspar et al., 2022), foot (Rogers, 1980) and eye preferences (Brown & Magat, 2011) in Australian parrots, left-leg preference for prey touching in spitting spiders (Ades & Ramires, 2002), right-leg preference in kicking undesirable males by female mosquitoes (Benelli et al., 2015), turning bias in ants (Hunt et al., 2014) and a higher frequency of being attacked on the right in trilobites (Babcock, 1993).

Ghirlanda et al. (2009) argued that population-level brain lateralisation can occur in two steps: first, individuals should benefit from increased cognitive efficiency by being lateralised in either direction (Güntürkün et al., 2000; Rogers et al., 2004; Vallortigara & Rogers, 2005); second, a population-level bias in preference to one direction should bring additional benefits, e.g. the majority of individuals moving in the same direction creates a dilution effect which reduces the chances of being eaten by predators (Ghirlanda & Vallortigara, 2004; Vallortigara & Rogers, 2005), while the minority may also enjoy a surprise advantage if predators learn which direction the majority of their prey prefer (Ghirlanda & Vallortigara, 2004). Although the additional benefits were first discussed in relation to prey–predator interactions, similar benefits might also emerge from intraspecific interactions. Ghirlanda et al. (2009) and Abrams & Panaggio (2012) have suggested that the population balance of right-handers vs. left-handers reflects the relative prevalence of cooperative vs. competitive interactions, with cooperative interactions promoting the fitness of the majority handedness type and competitive interactions promoting the fitness of the minority handedness type. All of these game theoretical models focus on social interactions, which are very likely to be mediated by genetic relatedness as shown in general cases of social evolution, yet our investigation is the first time that kin selection has been considered in human handedness. The predicted effect of relatedness on the evolution of handedness crucially depends on whether left-handedness is marginally altruistic or selfish. Although current data are not sufficient for answering that question, our analyses provide a framework within which future data can be motivated and conceptualised.

## Supporting information

Dong et al. supplementary material Dong et al. supplementary material

## Data Availability

All the equations and derivations can be found in our uploaded supplementary material.

## Supplementary material

To view supplementary material for this article, please visit https://doi.org/10.1017/ehs.2024.24

## Research transparency and reproducibility

All the equations and derivations can be found in our uploaded supplementary material.
